# Bringing gene therapy to regenerative medicine

**DOI:** 10.1016/j.omtm.2023.05.021

**Published:** 2023-06-16

**Authors:** Elizabeth R. Balmayor

**Affiliations:** 1 Experimental Orthopaedics and Trauma Surgery, Department of Orthopaedic, Trauma, and Reconstructive Surgery, RWTH Aachen University Hospital, 52074 Aachen, Germany

Gene therapy has its intellectual origins in the context of treating Mendelian disorders,^1^ with the fruits of its decades-long development reflected in the increasing number of gene therapeutics gaining regulatory approval. However, single-gene defects are rare. The potential scope of gene therapy was greatly broadened with the realization that it had potential application in common, complex conditions that lack a monogenetic basis. In this context, gene transfer serves as a drug delivery system for encoded therapeutic proteins and non-coding species of RNA. This application was first appreciated in the case of arthritis, and several arthritis gene therapies are now in clinical trials.^2^ This provided the impetus for expansion into the field of regenerative medicine.^3^

Regenerative medicine is an attractive application of gene therapy because most of the morphogens and growth factors of interest for this purpose are difficult to deliver to sites of tissue damage and have very short biological half-lives. Gene transfer offers the prospect of providing these proteins by local synthesis in a sustained fashion at concentrations sufficient to drive a robust regenerative process. Problems in bone healing provided the earliest application of this concept,^4^^,^^5^ which remains the area where most progress has been made.

Although bone is one of the few organs in the human body that can regenerate scarlessly, in about 5%–10% of cases, fractures fail to heal, leading to non-unions. These are recalcitrant clinical problems. The 1965 publication by Marshall Urist^6^ of a bone inductive extract with the ability to induce new endochondral bone formation revolutionized our understanding of bone biology. In the era preceding modern molecular technology, it took decades to isolate, purify, and eventually clone these molecules, which became known as bone morphogenetic proteins or BMPs.^7^ Once cloned, BMPs became available at scale, thereby enabling the first clinical use of BMPs in non-unions, which dates to the late 1980s.

Recombinant, human BMP-2 (rhBMP-2) is available for clinical use in the United States and Europe as part of a product known as Infuse™ Bone Graft, whose approved indications are high-energy open tibial fractures, spinal fusions, and certain oromaxillofacial applications. Additionally, it has wide off-label use^8^ in treating osseous non-unions and a related condition where a large segment of bone is missing, usually as a result of trauma. Beyond a certain critical size, segmental defects in bone never heal spontaneously and can lead to amputation.

Despite much promise, the clinical efficacy of rhBMP-2 has been disappointing. In attempts to improve outcomes, very high amounts of the protein are used, but these provoke numerous adverse side effects, some of them severe.^9^ Moreover, recent evidence suggests that bone formed in response to large amounts of rhBMP-2 is of inferior quality.^10^ Much of the blame for the mediocre clinical response to rhBMP-2 has focused on inadequate delivery from the collagen sponge used as a scaffold, and there has been considerable research into developing improved scaffolds that deliver higher amounts of rhBMP-2 locally for longer periods of time. Gene transfer was proposed as a superior technology for achieving this end.

Genetic delivery of BMP-2 indeed proved superior to protein delivery of rhBMP-2 in pre-clinical models of bone healing, but in an unexpected way—efficient healing via gene therapy occurred when only small amounts of BMP-2 were expressed *in vivo* for a short period of time.^11^ As described in this issue of *Molecular Therapy – Methods and Clinical Development*, Chris Evans and his team investigated why this was so.^12^

This *in vitro* study focused on BMP-2 signaling via SMAD phosphorylation using reporter murine mesenchymal cells where firefly luciferase expression is driven by elements of the Id1 promotor, an early response gene activated by BMP-2.^13^ Responses to BMP-2 synthesized endogenously as a result of transduction with adenovirus (Ad.BMP2) or lentivirus (LV.BMP2) were compared with those elicited by exogenously delivered rhBMP-2. The study confirmed the superiority of transgenic BMP-2 cDNA over rhBMP-2, showing that the former was at least 100-fold more effective in signaling. Interestingly, when analyzing the amount of BMP-2 produced by transduced cells, the authors found that over 30% of the BMP-2 remained cell associated; this was more pronounced (i.e., over 50%) when AdBMP2 was used. This was associated with partial resistance to noggin, a physiological inhibitor that binds to BMP-2. The secreted amounts of endogenously synthesized BMP-2 were very low, and media conditioned by the transduced cells expressing BMP-2 were unable to induce luciferase expression in naive reporter cells.

Co-culture experiments demonstrated that cells expressing BMP-2 were able to induce luciferase expression in adjacent naive cells. When BMP-2 cells were co-cultured with naive cells but separated by a permeable insert, there was no activation of the naive cells, suggesting that close proximity and possibly cell-to-cell contact was necessary for this to occur. To confirm that these data were not a result of using an established cell line, osteogenesis of primary cultures of human mesenchymal stromal cells was demonstrated.

In summary, the authors explain the superior activity of transgenic BMP-2 by its ability to elicit powerful autocrine and paracrine effects in a protected pericellular environment. Although the study focused on SMAD signaling via the BMP type 1A receptor, the authors left open the possibility of additional pathways, including intracrine signaling based on the observation that a substantial portion of transgenic BMP-2 remained cell associated.

These data provide mechanistic support for the use of gene therapy-based approaches to regenerating bone and provide, for the first time, a plausible explanation for the observation that gene transfer is superior to a bolus of protein in healing bone. The efficiency of tiny amounts of transiently expressed, endogenously synthesized BMP-2 in healing bone suggests novel opportunities beyond traditional gene therapy. Recently, messenger RNA (mRNA) has shown remarkable success in pre-clinical models of bone healing.^14^ A stable and non-immunogenic formulation of mRNA encoding BMP-2 efficiently healed critical-size bone defects in rats, with the regenerate rapidly achieving superior biomechanics and undergoing advanced tissue remodeling. Based on these encouraging findings, mRNA therapeutics promise to revolutionize the application of gene therapy to regenerative medicine by combining safety, efficacy, and affordability.^15^
